# Foreign Body Granuloma: A Diagnosis Not to Forget

**DOI:** 10.1155/2012/439836

**Published:** 2012-03-26

**Authors:** I. El Bouchti, F. Ait Essi, I. Abkari, M. Latifi, S. El Hassani

**Affiliations:** ^1^Rheumatology Department, Mohammed VI Medical Center, Marrakesh, Morocco; ^2^Department of Orthopedics and Orthopedic Surgery B, Mohammed VI Medical Center, Marrakesh, Morocco

## Abstract

Penetrating wounds of the foot are not uncommon. Many are caused by thorns or by fragments of wood that are retained in the foot, creating a foreign-body granuloma. The differential diagnosis for bony reaction to an unrecognised organic foreign body includes osteoid osteoma, chronic and acute osteomyelitis, tuberculosis granuloma, bone cyst, aneurysmal bone cyst, cortical fibrous defect, and neoplasm. We report the case of a boy suffering from a thorn inducing a lytic lesion of the fifth metatarsal that demonstrates the diagnosis difficulties of foreign body granuloma.

## 1. Introduction

Penetrating wounds of the foot are not uncommon. Many are caused by thorns or by fragments of wood that are retained in the foot, creating a foreign-body granuloma. Symptomatic lesions may develop years after the injury, and the patient may not remember a specific injury event. We report the case of a boy suffering from a thorn inducing a lytic lesion of the fifth metatarsal that demonstrates the diagnosis difficulties of foreign body granuloma.

## 2. Case Report

**Figure 1:**
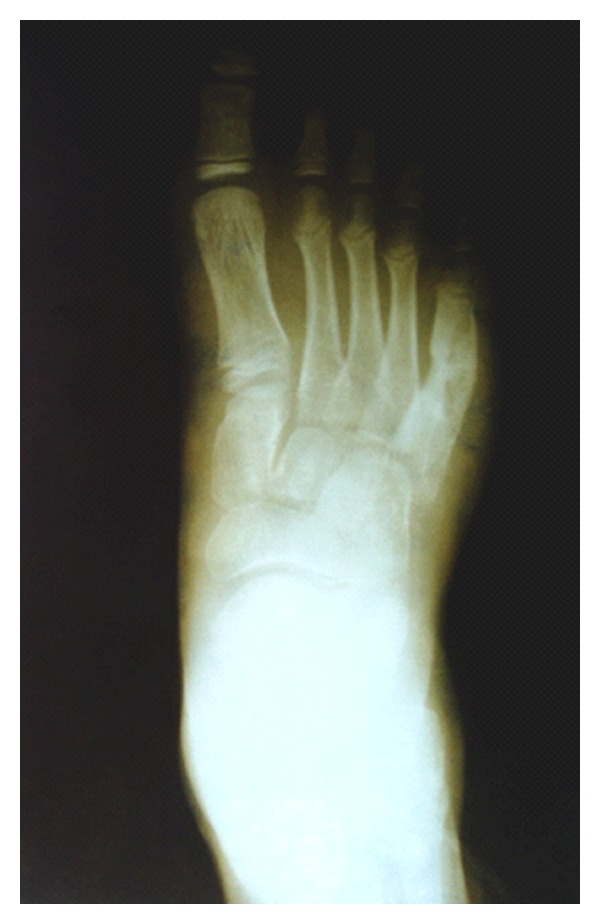
A radiograph of the right foot showed an eccentric lytic lesion of the cortex and the medullary space in the diaphysis of the fifth metatarsal with sclerotic margins.

**Figure 2:**
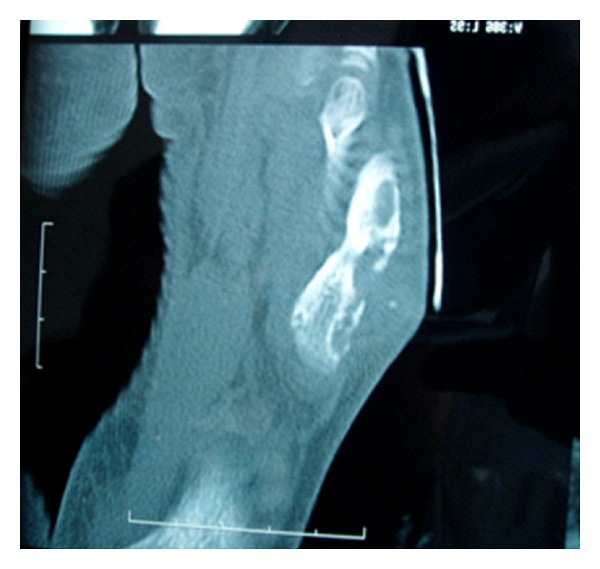
CT showed geodes with reactive sclerosis and soft tissue swelling without collection.

A 10-year-old boy presented with progressive swelling in the dorsolateral region of the right foot. He had had the symptoms for 3 years. He could recall no history of trauma. Physical examination showed a healthy patient with a tender mass on the dorsolateral region of the right foot measuring 3 cm/4 in diameter. The overlying skin was intact. There was neither tenosynovitis nor oedema. A radiograph of the right foot showed an eccentric lytic lesion of the cortex and the medullary space in the diaphysis of the fifth metatarsal with sclerotic margins (Figure 1). CT showed geodes with reactive sclerosis and soft tissue swelling without collection (Figure 2). The biopsy specimen revealed epithelio-gigantocellulary granuloma without caseous necrosis. He had a normal white-blood-cell count, a normal C-reactive protein level, and an erythrocyte sedimentation rate of 21 mm/hr. Tuberculosis research was negative (tuberculin skin test, sputum examination, and standard thoracic X-ray). Because of the suspicion of an osteolytic tumor, an open biopsy was performed. The cortex was found to be destroyed and replaced by granulation tissue. A thorn was found and removed. Histologically, chronic granulation tissue was confirmed. Six months after operation, radiographs show that the bone lesion was resolved, and remodeling occurred. 

## 3. Discussion

Retained foreign bodies in the feet following puncture wounds in children occur commonly. They include glass, metallic objects, and organic materials. Glass, ceramic, and metallic foreign bodies are almost always identified on plain radiographs. Organic materials and plastics, on the other hand, are diagnostic challenges because they do not show up on plain radiographic films [1]. The effects of plant thorn in soft and bony tissues include foreign body cyst, bursitis, tenosynovitis, synovitis, monoarthritis, and bone lesions that may mimic a tumor [2–4].

The most frequently reported injuries are those to the hands, knees, and feet (metatarsal, cuneiform, cuboid, and phalangeal lesions), and these injuries may be intra-articular or limited to the soft tissues [1, 5]. The median time from the injury to the detection of the osseous lesion is variable from some months to many years (4 months–20 years), and both the child and the family may have forgotten about the injury entirely. This can lead to a clinically significant delay in making the correct diagnosis [1, 3, 5]. If the foreign body is not removed immediately, or is not phagocytes during the acute inflammatory reaction, it becomes encapsulated with fibrous tissue and forms a granuloma [2]. The bone lesion that is induced by a thorn or a wood splinter usually appears to be a consequence of infection resulting in osteolysis or periostitis or both [5].

If a history of antecedent skin puncture is not recognized and if the foreign body is radiolucent, the radiograph appearance of the bone reaction can be confusing and can even mimic a neoplasm. The others differential diagnosis for bony reaction to an unrecognised organic foreign body includes osteoid osteoma, chronic and acute osteomyelitis, tuberculosis granuloma, bone cyst, aneurysmal bone cyst, and cortical fibrous defect [2, 3].

Retained nonradiopaque foreign body inside soft tissue can be a cause of prolonged morbidity. Detection and localization is a difficult task with conventional radiography. Ultrasonography, computed tomography (CT), and magnetic resonance imaging (MRI) are other modes of evaluation, but both of CT and MRI are expensive and not easily available [1, 6]. Splinters that have been soaked for less than three days or those that are located near the bone are not detected reliably with any imaging method [5]. Ultrasonography is sensitive and specific for detection and localization of foreign body which should be included in evaluation for clinically suspicious retained nonradiopaque foreign body in soft tissue of extremities [6]. Excision of the foreign body allows symptomatic and radiographic cure.

## 4. Conclusion

Even in the absence of a definite history of trauma, an organic foreign body lesion should be considered in the differential diagnosis of a lytic lesion of bone, so that unnecessary delays and potentially dangerous treatment can be avoided. Surgical exploration of a granuloma must include the deeper tissue planes so that a small thorn is not missed.
